# Fungal cytochrome P450 database

**DOI:** 10.1186/1471-2164-9-402

**Published:** 2008-08-28

**Authors:** Jongsun Park, Seungmin Lee, Jaeyoung Choi, Kyohun Ahn, Bongsoo Park, Jaejin Park, Seogchan Kang, Yong-Hwan Lee

**Affiliations:** 1 Fungal Bioinformatics Laboratory, Seoul National University, Seoul 151-921, Korea; 2 Department of Agricultural Biotechnology, Seoul National University, Seoul 151-921, Korea; 3 Center for Fungal Genetic Resource, Seoul National University, Seoul 151-921, Korea; 4 Department of Plant Pathology, The Pennsylvania State University, University Park, PA 16802, USA; 5 Center for Agricultural Biomaterials, Seoul National University, Seoul 151-921, Korea

## Abstract

**Background:**

Cytochrome P450 enzymes play critical roles in fungal biology and ecology. To support studies on the roles and evolution of cytochrome P450 enzymes in fungi based on rapidly accumulating genome sequences from diverse fungal species, an efficient bioinformatics platform specialized for this super family of proteins is highly desirable.

**Results:**

The Fungal Cytochrome P450 Database (FCPD) archives genes encoding P450s in the genomes of 66 fungal and 4 oomycete species (4,538 in total) and supports analyses of their sequences, chromosomal distribution pattern, and evolutionary histories and relationships. The archived P450s were classified into 16 classes based on InterPro terms and clustered into 141 groups using tribe-MCL. The proportion of P450s in the total proteome and class distribution in individual species exhibited certain taxon-specific characteristics.

**Conclusion:**

The FCPD will facilitate systematic identification and multifaceted analyses of P450s at multiple taxon levels via the web. All data and functions are available at the web site .

## Background

Cytochrome P450 is the collective name for a super family of heme-containing monooxygenases. P450 enzymes not only participate in the production of diverse metabolites but also play critical roles in organism's adaptation to specific ecological and/or nutritional niches by modifying potentially harmful environmental chemicals. In fungi, P450 enzymes have contributed to exploration of and adaptation to diverse ecological niches [1,2].

Rapidly accumulating genome sequences from diverse fungal species, including more than 80 species with more currently being sequenced [3], offer opportunities to study the genetic and evolutionary mechanisms underpinning different fungal life styles at the genome level [4-7]. To support such studies with the focus on cytochrome P450s, we constructed a new platform named as the Fungal Cytochrome P450 Database (FCPD), which archives P450s in most sequenced fungal and oomycetes species and allows comparison of the archived data with previously published datasets, such as the Cytochrome P450 Engineering Database [8], a manually curated P450 database at  (referred as the Nelson's P450 database herein), and P450 datasets derived from extensive phylogenetic analyses of selected fungal taxon groups [9,10]. The FCPD also supports multifaceted analyses of P450s using various web-based bioinformatics tools supported by the Comparative Fungal Genomics Platform (CFGP; ) [3]. The FCPD, in combination with high-throughput experimental approaches, will advance our understanding of the roles and evolution of P450s.

## Construction and content

### Pipeline for identifying and classifying fungal P450s

**Figure 1:**
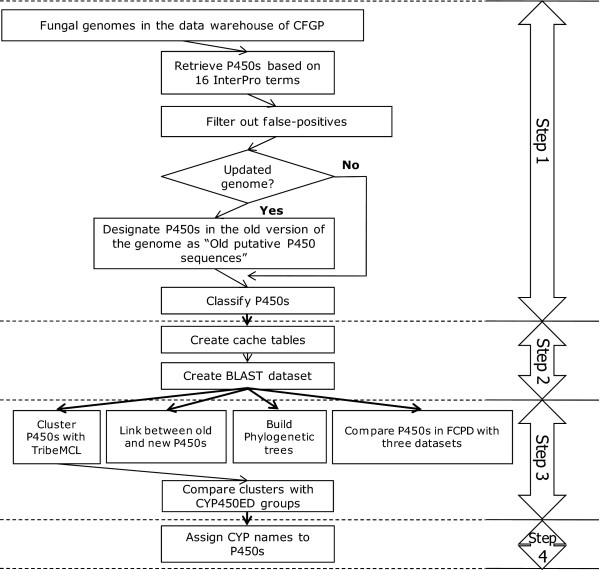
**Data retrieval pipeline in FCPD**. Four-steps involved in identifying and classifying fungal P450s in FCPD are presented as a flowchart.

**Figure 2:**
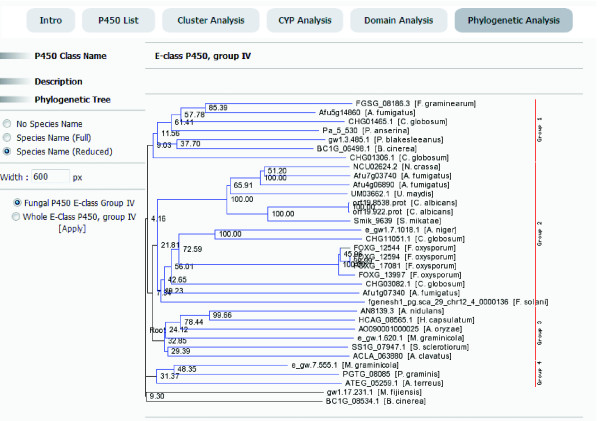
**Phylogenetic analysis of E-class P450, group IV**. A bootstrapped phylogenetic tree was constructed using Phyloviewer . Four different clades in the tree are indicated as blue lines.

To identify P450 proteins from genome sequences, standardized genome databases managed by CFGP () [3] and annotated information of each ORF by InterPro scan [11] were used. The pipeline for the identification and archiving of P450s consists of four steps (Figure 1). In the first step, all proteins carrying one or more of 16 InterPro terms associated with cytochrome P450 were identified and classified according to associated InterPro terms. Domain information of P450 proteins was also retrieved from the InterPro scan results. To filter out potential false positives (i.e., those carrying a very short domain), the minimum length for IPR001128 (Cytochrome P450) was set at 25 amino acid (aa). Since some of these potential false positives might indeed belong to novel P450s, rather than discarding them, they were labelled as "questionable P450" in FCPD. Secondly, using the collection of putative P450 sequences, cache tables, especially for results from several statistical analyses, were created to speed up data retrieval. BLAST datasets were also generated to support BLAST searches of P450s via the FCPD web site and cluster analysis. Thirdly, class-specific and cluster-specific neighbour joining phylogenetic trees that show relationships among P450s within individual phylogenetic groups (e.g., Figure 2) were constructed (bootstrapped with 2,000 or 10,000 repeats), which are displayed by Phyloviewer (; Park *et al*., unpublished) on the FCPD web site. Using the BLAST dataset, fungal P450s were clustered using tribe-MCL [12], and compared with the data in three publicly available databases: the Cytochrome P450 Engineering database [8], the Nelson's P450 database, and a set of phylogenetically analyzed P450s in multiple fungal species [9,10]. Results from this comparison were stored in the FCPD for viewing via the FCPD web site. For species with multiple versions of genome annotation, data generated using different versions were linked to provide the history of annotation.

**Figure 3:**
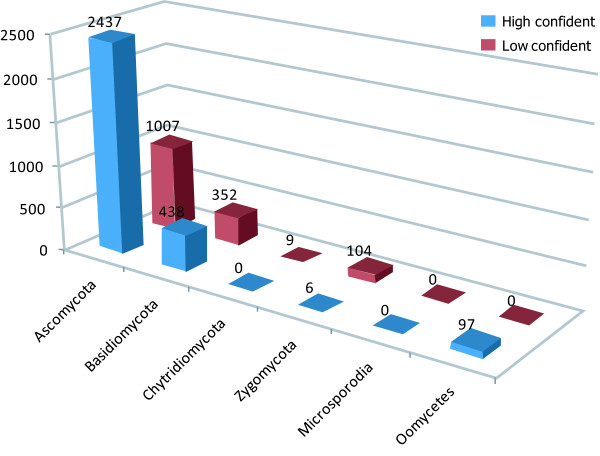
**Confidence levels in the family assignment of individual P450s in different fungal phyla**. Five fungal phyla and oomycetes are shown below the X-axis. The Y-axis indicates the proportion of P450s classified with high confidence or low confidence. The numbers on the top of each bar indicate the number of P450 in each class.

As the fourth step, using BLAST all P450s archived in FCPD were matched to the corresponding families in the Nelson's P450s database, which contains manually curated data based on the P450 International Nomenclature [13,14]. For each P450, the assigned family name was considered highly confident ('> = 44% identity' in the site), when the degree of aa sequence identity was 44% or higher. When no match at that level could be found in the Nelson's P450 database, the best hit in BLAST search was chosen to assign the family name and labelled as low confidence ('< 44% identity' in the site). Considering that P450s are very diverse and that the Nelson's P450 database covers less fungal species than FCPD, it is highly likely that some of the P450s with low confidence represent novel families that have yet to be registered in the Nelson's P450 database (Figure 3). This annotation result was stored in FCPD and can be viewed through the FCPD web site.

In the genomes of 66 fungal and 4 oomycete species, 4,538 putative P450 genes were identified. Although oomycete species belong to the kingdom Stramenophila and show closer phylogenetic relationships to brown algae and diatoms [15], they have been traditionally studied by mycologists due to their morphological similarities with true fungi, and their P450s were included in FCPD.

### Evaluation of the accuracy of annotation via the automated pipeline in FCPD by comparing with data archived in the manually curated Nelson's P450s database

The automated annotation process of P450 in FCPD may result in some false-positives and negatives. To evaluate its accuracy, all 886 P450s identified using the pipeline in 12 fungal species were compared with manually curated data in the Nelson's P450 database. The positive predictive value (PPV; the proportion of the predicted P450s in FCPD to P450s that have been archived in the Nelson's P450 database) was 0.894 (792 out of 886 P450s in FCPD were matched to P450s in Nelson's P450 database). Some putative false positives in FCPD appeared to be pseudo genes. Another factor that contributed to the discrepancy between the two sources is that some data in the Nelson's P450 database were based on a version earlier than what was used for FCPD (e.g., version 4 of *Magnaporthe oryzae *genome having been used for the former, while FCPD being based on version 5). Gene prediction models employed to analyze different versions might have had different predictions. In contrast, 1,032 out of 1,034 fungal P450s curated in the Nelson's P450 database were identified as P450 by the FCPD pipeline (99.8% sensitivity), supporting the reliability of the FCPD pipeline. The two P450s not identified as P450 by FCPD came from *Phytophthora sojae *and *P. ramorum*, respectively and corresponded to truncated sequences (34 and 89 aa, respectively, and were labelled as fragment of P450 in the Nelson's P450 database). Detailed analyses of the underlying reasons for the inconsistency between the two sources will help us improve the automated annotation pipeline of FCPD.

### Notable features in fungal P450s in the taxonomic context

The numbers of P450s in individual species exhibited certain taxon-specific features (Table 1). Within the phylum Ascomycota, members of the subphylum Pezizomycotina typically carry around 100 P450s with the exception of four species (*Coccidioides immitis*, *Histoplasma capsulatum*, *Uncinocarpus reessi *and *Neurospora crassa*) that only carry 22 to 46 P450s. The proportion of P450s in the total proteome in the subphylum Pezizomycotina (0.63% in average) is twice as large as that of vertebrates (0.33%) but is less than that of plant species (0.82%). In contrast to the Pezizomycotina, species in the subphyla Saccharomycotina and Taphrinomycotina have a very few P450s (e.g., only 3 P450s in *Saccharomyces cerevisiae *and 2 P450s in *Schizosaccharomyces pombe*). Within the phylum Basidiomycota, *Postia placenta *carries 353 P450s (2.06% of the total proteome), while strains of *Cryptococcus neoformans *have 5 to 6 P450s (0.08% ~ 0.09% of the total proteome). Interestingly, *Encephalitozoon cuniculi *and *Antonospora locustae*, species in the phylum Mycosporodia, do not appear to have any P450s, probably reflecting their obligate, intracellular parasitic life style. Four oomycete species, including *Phytophthora infestans*, *P. sojae*, *P. ramorum *and *Hyaloperonospora parasitica*, also carry relatively low numbers of P450s (9 to 35 and 0.06 to 0.2% of the total proteome).

**Table 1:** P450s in the fungal kingdom

**Species^a^**	**# of ORFs**	**# of P450s**	**Ratio (%)**	**Source^d^**	**Ref**
**Fungi (Kingdom)**					
**Ascomycota (Phylum)**					
**Pezizomycotina (Subphylum)**					
*Botrytis cinerea*	16,448	136	0.83	BI	-
*Sclerotinia sclerotiorum*	14522	96	0.66	BI	-
*Aspergillus clavatus*	9,121	97	1.06	BI	[15,16]
*Apsergillus flavus*	12,604	159	1.26	BI	[17]
*Aspergillus fischerianus*^c^	10,403	99	0.95	BI	[16]
*Aspergillus fumigatus *A1163	9,929	76	0.77	TIGR	[16]
*Aspergillus fumigatus *Af293	9,887	80	0.81	TIGR	[18]
*Aspergillus nidulans*	10,701	122	1.14	BI	[19]
*Aspergillus niger *ATCC1015	11,200	154	1.38	JGI	-
*Aspergillus niger *CBS513.88	14,086	151	1.07	NCBI	[20]
*Aspergillus oryzae*	12,063	163	1.35	DOGAN	[21]
*Aspergillus terreus*	10,406	125	1.20	BI	[15]
*Coccidioides immitis *RS	10,457	45	0.43	BI	-
*Coccidioides immitis *H538.4	6,991	44	0.63	BI	-
*Coccidioides immitis *RMSCC 2394	7,162	44	0.61	BI	-
*Coccidioides immitis *RMSCC 3703	6,935	43	0.62	BI	-
*Histoplasma capsulatum *G186AR	7,454	22	0.30	WGSC	-
*Histoplasma capsulatum *G217B	8,038	46	0.57	WGSC	-
*Histoplasma capsulatum *NAm1	9,349	42	0.45	BI	-
*Uncinocarpus reesii*	7,798	41	0.53	BI	-
*Chaetomium globosum*^c^	11,124	92	0.83	BI	-
*Fusarium graminearum *PH-1	13,321	118	0.89	BI	[22]
*Fusarium graminearum *GZ3639^b^	6,694	45	0.67	BI	[22]
*Fusarium oxysporum*	17,608	170	0.97	BI	-
*Fusarium verticillioides*	14,199	129	0.91	BI	-
*Fusarium solani*	15,707	162	1.03	JGI	-
*Magnaporthe oryzae*	12,841	139	1.08	BI	[23]
*Neurospora crassa*	9,842	43	0.40	BI	[24]
*Podospora anserine*	10,596	115	1.09	IGM	[25]
*Trichoderma reesei*	9,129	73	0.80	JGI	[26]
*Trichoderma virens*	11,643	120	1.03	JGI	-
*Mycosphaerella graminicola*	11,395	81	0.71	JGI	-
*Mycosphaerella fijiensis*	10,313	94	0.91	JGI	-
*Stagonospora nodorum*	16,597	153	0.92	BI	[27]
**Saccharomycotina (Subphylum)**					
*Candida albicans *SC5314	6,090	10	0.16	SGTC	[28,29]
*Candida albicans *WO-1	6,157	10	0.16	BI	-
*Candida dubliniensis*	6,027	10	0.17	SI	-
*Candida glabrata*	5,165	3	0.06	CBS	[30]
*Candida guilliermondii*	5,920	10	0.17	BI	-
*Candida lusitaniae*	5,941	9	0.15	BI	-
*Candida parasilosis*	5,733	14	0.24	BI	-
*Candida tropicalis*	6,258	12	0.19	BI	-
*Debaryomyces hansenii*	6,354	9	0.14	CBS	[30]
*Ashbya gossypii*	4,717	3	0.06	NCBI	[31]
*Kluyveromyces lactis*	5,327	5	0.09	GS	[30]
*Kluyveromyces polysporus*	5,367	4	0.07	SIG	[32]
*Kluyveromyces waltii*	4,935	3	0.06	BI	[33]
*Lodderomyces elongisporus*	5,796	10	0.17	BI	-
*Pichia stipitis*	5,839	10	0.17	JGI	[34]
*Saccharomyces bayanus *MCYC 623	9,385	3	0.03	BI	[33]
*Saccharomyces bayanus *623-6C YM4911	4,966	3	0.06	WGSC	[35]
*Saccharomyces castellii*	4,677	3	0.06	VBI	[35]
*Saccharomyces cerevisiae *S288C	5,898	3	0.06	SGD	[36]
*Saccharomyces cerevisiae *RM11-1a	5,366	3	0.06	BI	-
*Saccharomyces cerevisiae *YJM789	5,903	3	0.05	SI	[37]
*Saccharomyces kudriavzevii*	3,768	3	0.08	VBI	[33]
*Saccharomyces kluyveri*	2,968	3	0.10	WGSC	[35]
*Saccharomyces mikatae*	9,016	3	0.03	BI	[33]
*Saccharomyces mikatae*	3,100	1	0.03	WGSC	[35]
*Saccharomyces paradoxus*	8,939	3	0.03	BI	[33]
*Yarrowia lipolytica*	6,524	17	0.26	CBS	[30]
**Taphrinomycotina (Subphylum)**					
*Pneumocystis carinii*^b,c^	4,062	2	0.05	SI	-
*Schizosaccharomyces pombe*	5,005	2	0.04	GeneDB	[38]
*Schizosaccharomyces japonicus*	5,172	2	0.04	BI	-
**Basidiomycota (Phylum)**					
**Agricomycotina (Subphylum)**					
*Postia placenta*	17,173	353	2.06	JGI	-
*Phanerochaete chrysosporium*	10,048	145	1,14	JGI	[39]
*Coprinus cinereus*	13,544	138	1.02	BI	-
*Laccaria bicolor*	20,614	91	0.44	JGI	[40]
*Cryptococcus neoformans *Serotype A	7,302	6	0.08	BI	-
*Cryptococcus neoformans *Serotype B	6,870	6	0.09	NCBI	-
*Cryptococcus neoformans *Serotype D B3501-A	6,431	5	0.08	SGTC	[41]
*Cryptococcus neoformans *Serotype D JEC21	6,475	5	0.08	SGTC	[41]
**Pucciniomycotina (Subphylum)**					
*Sporobolomyces roseus*	5,536	7	0.13	JGI	-
*Puccinia graminis*	20,567	18	0.09	BI	-
**Ustilaginomycotina (Subphylum)**					
*Malassezia globosa*	4,286	6	0.14	PGC	[42]
*Ustilago maydis *521	6,689	21	0.33	BI	[43]
*Ustilago maydis *FB1	6,950	21	0.30	BI	[43]
**Chytridiomycota (Phylum)**					
*Batrachochytrium dendrobatidis *JEL423	8,818	9	0.10	BI	-
**Mucoromycotina (Subphylum *incertae sedis*)**					
*Rhizopus oryzae*	17,467	50	0.29	BI	-
*Phycomyces blakesleeanus*	14,792	56	0.38	JGI	-
**Microsporidia (Phylum)**					
*Antonospora locustae*^b,c^	2,606	0	0.00	JBPC	-
*Encephalitozoon cuniculi*	1,996	0	0.00	GS	[44]
**Stramenopila (Kingdom)**					
**Peronosporomycota (Phylum)**					
*Phytophthora infestans*^c^	22,658	29	0.13	BI	-
*Phytophthora sojae*	19,276	35	0.18	JGI	[45]
*Phytophthora ramorum*	16,066	33	0.21	JGI	[45]
*Hyaloperonospora parasitica*	14,789	9	0.06	VBI	-

**Total**	797,891	4,538	0.57		

^**a**^List of species archived in FCPD sorted by a recently established taxonomic scheme [46].

^**b**^These genomes have not been completed sequenced.

^**c**^Insufficient exon/intron information

^**d**^SGTC, Stanford Genome Technology Center; SI, Sanger Institute; CBS, Center For Biological Sequences; BI, Broad Institute; WGSC, Washington University Genome Sequencing Center; JGI, DOE Joint Genomic Institute; DOGAN, Database Of the Genomes Analyzed at Nite; GS, Genoscope; VGI, Virginia Bioinformatics Institute; BCM, Baylor College of Medicine; JBPC, Josephine Bay Paul Center for Comparative Molecular Biology and Evolution; SIG, Trinity College Dublin, Smurfit Institute of Genetics; IGM, Instituté de Génétique et Microbiologie; PGC, Procter & Gamble Co.

Three P450 classes defined by InterPro terms, including group I in E-class P450, group IV in E-class P450 and Cytochrome P450, contain 3,866 out of 4,538 (85.2%) fungal/oomycete P450s. Only 8 out of 16 classes have fungal/oomycete P450s. Among other classes, P450s belonging to the pisatin demethylase (PDA)-like class are present only in the subphylum Pezizomycotina (phylum Ascomycota) and in the phylum Basidiomycota, suggesting the possibility that PDA-related P450s might have emerged twice independently during fungal evolution.

### Distribution patterns of fungal P450s among clusters and clans

**Figure 4:**
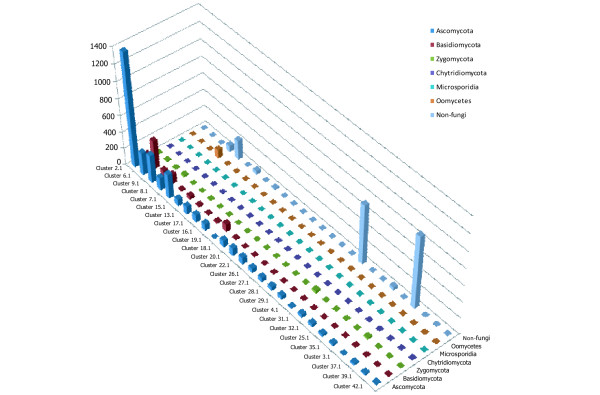
**Distribution pattern of 25 major P450 clusters**. Cluster names are shown below the X-axis, and the names of fungal phyla are shown at the y-axis. Non-fungi indicate P450s in plants and animals. The Z-axis indicates numbers of P450s in individual groups.

When fungal/oomycetes P450s were combined with 5,447 P450s extracted from 40 other eukaryotic and prokaryotic species and clustered using tribe-MCL (with inflation factor of 5.0; the most strict condition for clustering based on sequence similarity), 141 clusters were identified. Among these, 74 clusters contain only fungal P450s, suggesting that many fungal P450s have a configuration unique to fungi. The taxonomic origins of fungal P450s in the 26 clusters that contain more than 10 fungal P450s were analyzed (Figure 4). P450s in the phylum Ascomycota are dominant because of abundant genome sequences from members of this group. Cluster 19.1 is dominated by P450s encoded members of the subphylum Agricomycotina (phylum Basidiomycota) and Clusters 3.1 and 4.1 are Zygomycota-specific. Cluster 8.1 contains 101 out of 106 oomycetes P450s (95.3%). Nine P450s encoded by *Batrachochytrium dendrobatidis*, the only sequenced species in the phylum Chitridiomycota, are scattered to 8 clusters, suggesting that they likely have distinct functions and evolutionary origins. Sequences of additional genomes are needed to further investigate the evolution of P450s in this phylum.

To compare the relationship between P450 clusters and clans, 115 clans identified in four species (including 375 P450s in total), including *M. oryzae*, *Fusarium graminearum*, *N. crassa *and *Aspergillus nidulans *[9], were collected and analyzed. Interestingly, only 4 out of 115 clans (6.1%) are scattered to more than one P450 clusters. For example, P450s included in clan FF59 were distributed to four P450 clusters (Clusters 4.1, 8.1, 31.1 and 73.1). However, each of the remaining clans belongs to one specific cluster, supporting a good correlation between two classification systems.

### Assignment of P450s archived in FCPD to individual P450 families based on the international nomenclature scheme

The Nelson's P450 database classified 1,016 (98.26%) out of 1,034 fungal/oomycete P450s into 276 P450 families. Most P450s in FCPD (4,446 out of 4,538; 97.97%) were matched to corresponding families in the Nelson's P450 database (see above). 2,978 P450s (66.98%) were tagged to specific families with high confidence, while 1,468 P450s (33.02%) were assigned to families with low confidence (Figure 3). In the phylum Ascomycota, the assignment of 1,007 P450s (29.24%) was supported with low confidence. In the phylum Basidiomycota, the proportion was 44.56% (352 out of 790 P450s). More than 90% P450s (104 out of 110) in the phylum Zygomycota and 100% P450s in the phylum Chytridiomycota did not closely match with any families in the Nelson's P450 database. These results strongly suggest that new fungal families need to be defined.

### Update of FCPD

Considering the rapid increase in fungal genome sequencing [3], timely update of FCPD is critical to present the latest information to users. The BLAST dataset, bootstrapped phylogenetic trees specific for individual classes and clusters, results from clustering analysis and annotation of P450s based on the international P450 nomenclature will be updated automatically once new P450s have been identified via the identification pipeline. Since the identification of P450s depends on the accuracy of a gene model employed to annotate the genome, as a new version of previously released genome sequences becomes available, FCPD will be updated with the data based on earlier versions being tagged as an "Old putative P450 sequences." Links between new and old versions will be provided.

## Utilities and discussion

### Accessing lists and sequences of fungal P450s based on species of origin and taxonomic position

To support efficient search and retrieval of sequences of P450s, data archived in FCPD can be browsed and searched through multiple methods. Upon selecting a species of interest, general information about the species and a list of its P450s can be viewed. From this list, any P450 sequences can be stored in a personal data repository called the Favorite, in which six useful bioinformatic tools can be utilized to analyze the stored data. The Favorite is a virtual space for storing sequences archived in CFGP [3]. A list of P450s belonging to each class defined by InterPro terms or cluster can also be displayed. Taxonomical distribution of P450s, resulted from comparison with data in the Cytochrome P450 Engineering Database (CYP450ED) [8] and two previous studies on fungal P450s [9,10], can be browsed. P450 sequences in FCPD can also be searched by gene name.

### BLAST search of all or subsets of P450s

In FCPD, five different databases of P450s, including all P450s (including those from plants and animals), all fungal/oomycete P450s and three fungal phylum-specific databases of P450s, can be searched using BLAST. Additionally, fungal P450 sequences in the Nelson's P450 database can also be searched. From BLAST search results, sequences of individual P450s can be saved in the Favorite for subsequent analyses.

### Analyses of P450s using tools in the Comparative Fungal Genomics Platform

Many on-line databases that archive gene families allow downloading of all or part of data to user's computer but often do not provide data analysis tools via the database site. Consequently, to conduct desired analyses, users may have to visit multiple websites to access desired data analysis tools and/or install programs in personal computer. In FCPD, sequences of one or more fungal P450s can be selected by clicking check boxes next to each P450 and stored them into the Favorite. The Object Browser in FCPD supports the transfer of chosen sequences from the Favorite to CFGP in which the data can be analyzed using six useful bioinformatics tools [3]. These tools include BLAST, ClustalW, InterPro Scan, PSort, SignalP 3.0 and BLASTMatrix. The BLASTMatrix is a novel tool for surveying the presence of genes homologous to a query in multiple species simultaneously. Once any new analysis tool has been added to CFGP, users of FCPD will be able to use the tool immediately.

### Visualization of chromosomal distribution patterns of P450s via SNUGB

**Figure 5:**
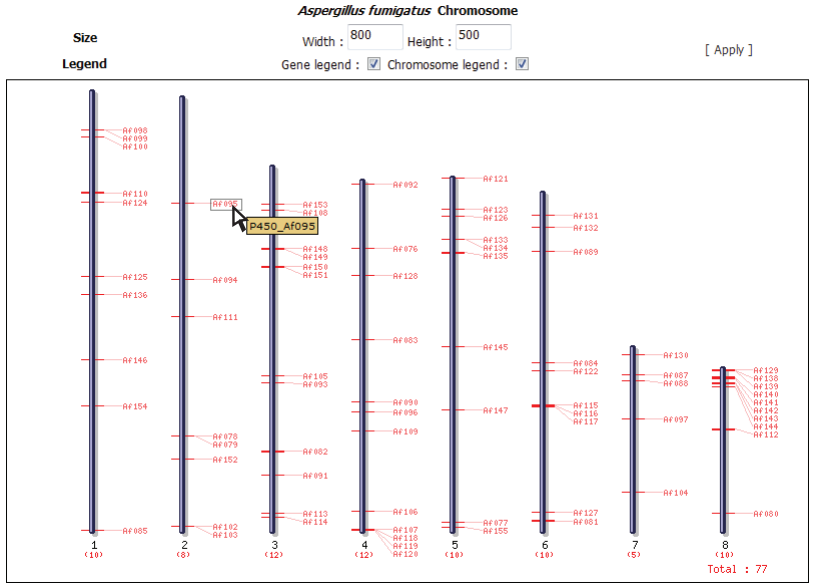
**Chromosomal distribution of P450s on the genome of *Aspergillus fumigatus***. On eight chromosomes of *A. fumigatus*, P450s identified in FCPD were displayed as red bars with their names. When mouse cursor moves on each name, a yellowish label will appear, which provides link to information page of chosen P450. This display is supported by SNUGB .

To aid for the visualization of chromosomal distribution pattern of P450s for species with available physical chromosome map information, FCPD provides a diagram illustrating position of P450s on individual chromosomes (Figure 5), which are drawn by a newly developed genome browser called SNUGB (; Jung *et al.*, submitted). Currently, chromosomal maps of 13 fungal species are available.

## Conclusion

To our knowledge, FCPD is the most comprehensive database that archives and classifies P450s in publicly available fungal and oomycete genomes (65 fungal and 4 oomycete species) through a systematic identification pipeline. The reliability of the pipeline in retrieving fungal P450 sequences was evaluated by comparing resulting data with other established datasets, and the data from these sources were archived in FCPD for comparison and search. The pipeline also links annotated information from different versions of fungal genome sequences. Numbers of P450s in individual fungal species vary widely, and fungal specific P450 clusters were found via clustering analysis. In combination with other bioinformatic platforms, such as CFGP [3], Phyloviewer (; Park *et al.*, unpublished), and SNUGB (; Jung *et al.*, submitted), FCPD provides a highly integrated platform supporting systematic studies on fungal P450s.

## Availability and requirements

All data described in this paper can be freely browsed and downloaded through the FCPD web site at .

## Authors' contributions

JSP, SK and Y–HL designed FCPD and wrote the manuscript. SL, KA, JC and BP developed the web site of FCPD. JSP, JC and BP developed the functionalities of FCPD supported by CFGP, Phyloviewer and SNUGB. JJP designed the FCPD web site.
